# 色谱技术在抗体分离纯化中的应用进展

**DOI:** 10.3724/SP.J.1123.2023.12010

**Published:** 2024-06-08

**Authors:** Jiawei LIU, Changwei TANG, Yiran XIA, Quan BAI

**Affiliations:** 合成与天然功能分子教育部重点实验室, 西北大学现代分离科学研究所, 陕西省现代分离科学重点实验室, 西北大学化学与材料科学学院, 陕西 西安 710127; Key Laboratory of Synthetic and Natural Functional Molecule of the Ministry of Education, Institute of Modern Separation Science, Key Laboratory of Modern Separation Science in Shaanxi Province, College of Chemistry & Materials Science, Northwest University, Xi'an 710127, China

**Keywords:** 抗体, 色谱技术, 分离纯化, 亲和色谱, 混合模式色谱, 温敏色谱, 综述, antibody, chromatographic techniques, separation and purification, affinity chromatography, mixed-mode chromatography, temperature-responsive chromatography, review

## Abstract

抗体作为一类重要的生物药物,在疾病诊断和治疗等方面拥有广阔的前景。抗体药物的需求逐年上升,生产规模不断扩大,下游抗体纯化已经成为抗体药物生产的瓶颈。色谱技术具有选择性好、分离效率高等优点,在抗体纯化领域占据主导地位。抗体分离纯化流程一般包括样品的粗提、粗提物精制和抗体精纯3个步骤,每个步骤都涉及色谱分离技术。目前已有多种色谱技术(如亲和色谱、离子交换色谱、疏水作用色谱、混合模式色谱以及新型的温敏色谱等)应用于抗体的分离纯化。本文介绍了近年来国内外各种色谱技术在抗体分离纯化方面所取得的研究进展,并对抗体色谱分离技术的未来发展趋势进行了展望。

## Abstract (en)

Antibody drugs are becoming increasingly popular in disease diagnosis, targeted therapy, and immunoprevention owing to their characteristics of high targeting ability, strong specificity, low toxicity, and mild side effects. The demand for antibody drugs is steadily increasing, and their production scale is expanding. Upstream cell culture technology has been greatly improved by the high-capacity production of monoclonal antibodies. However, the downstream purification of antibodies presents a bottleneck in the production process. Moreover, the purification cost of antibodies is extremely high, accounting for approximately 50%-80% of the total cost of antibody production. Chromatographic technology, given its selectivity and high separation efficiency, is the main method for antibody purification. This process usually involves three stages: antibody capture, intermediate purification, and polishing. Different chromatographic techniques, such as affinity chromatography, ion-exchange chromatography, hydrophobic interaction chromatography, mixed-mode chromatography, and temperature-responsive chromatography, are used in each stage. Affinity chromatography, mainly protein A affinity chromatography, is applied for the selective capture and purification of antibodies from raw biofluids or harvested cell culture supernatants. Other chromatographic techniques, such as ion-exchange chromatography, hydrophobic interaction chromatography, and mixed-mode chromatography, are used for intermediate purification and antibody polishing. Affinity biomimetic chromatography and hydrophobic charge-induction chromatography can produce antibodies with purities comparable with those obtained through protein A chromatography, by employing artificial chemical/short peptide ligands with good selectivity, high stability, and low cost. Temperature-responsive chromatography is a promising technique for the separation and purification of antibodies. In this technique, antibody capture and elution is controlled by simply adjusting the column temperature, which greatly eliminates the risk of antibody aggregation and inactivation under acidic elution conditions. The combination of different chromatographic methods to improve separation selectivity and achieve effective elution under mild conditions is another useful strategy to enhance the yield and quality of antibodies. This review provides an overview of recent advances in the field of antibody purification using chromatography and discusses future developments in this technology.

抗体(antibody)是由抗原刺激而产生的一类具有免疫功能的蛋白质。抗体能够特异性识别与清除进入宿主体内的细菌、病毒等病原体,是机体免疫系统的重要组成部分,存在于哺乳动物的体液(血液或淋巴液)内和B细胞表面^[1,2]^。抗体不仅可以直接参与外源物质的清除,还可以触发其他非特异性效应功能,如抗体依赖细胞介导的细胞毒性作用(ADCC效应)、补体依赖的细胞毒性作用(CDC效应)以及抗体依赖细胞介导的吞噬作用(ADCP效应)等^[3]^,产生免疫治疗的功效。因此抗体药物具有靶向性高、特异性强、毒副作用低等特点,在疾病诊断、靶向治疗、免疫预防等方面得到了广泛的应用^[4,5]^。

抗体有多克隆、单克隆和基因工程抗体等多种类型。在动物血浆或血清中存在的抗体成分也称为免疫球蛋白(immunoglobulin)。免疫球蛋白的基本结构单元是一个Y型的异源四肽链,各肽链之间由二硫键连接,如图1所示。其中分子质量较大(50 kDa)的两条链称为重链(heavy chain, H),有*α*、*γ*、*δ*、*ε*和*μ* 5种亚型,对应的免疫球蛋白分别为IgA、IgG、IgD、IgE和IgM。分子质量较小(25 kDa)的两条链称为轻链(light chain, L),有*κ*和*λ* 2种亚型^[3,5]^。抗体包括两个功能结构域,分别是两个抗原结合区域(Fab段)和一个可结晶片段(Fc段)。其中Fab段可识别和结合抗原,其N端属于可变区,决定了抗体的抗原结合特异性,而Fc段则是免疫球蛋白与效应分子或者细胞相互作用的部位,其重链亚型决定了抗体的种类。

**图 1:**
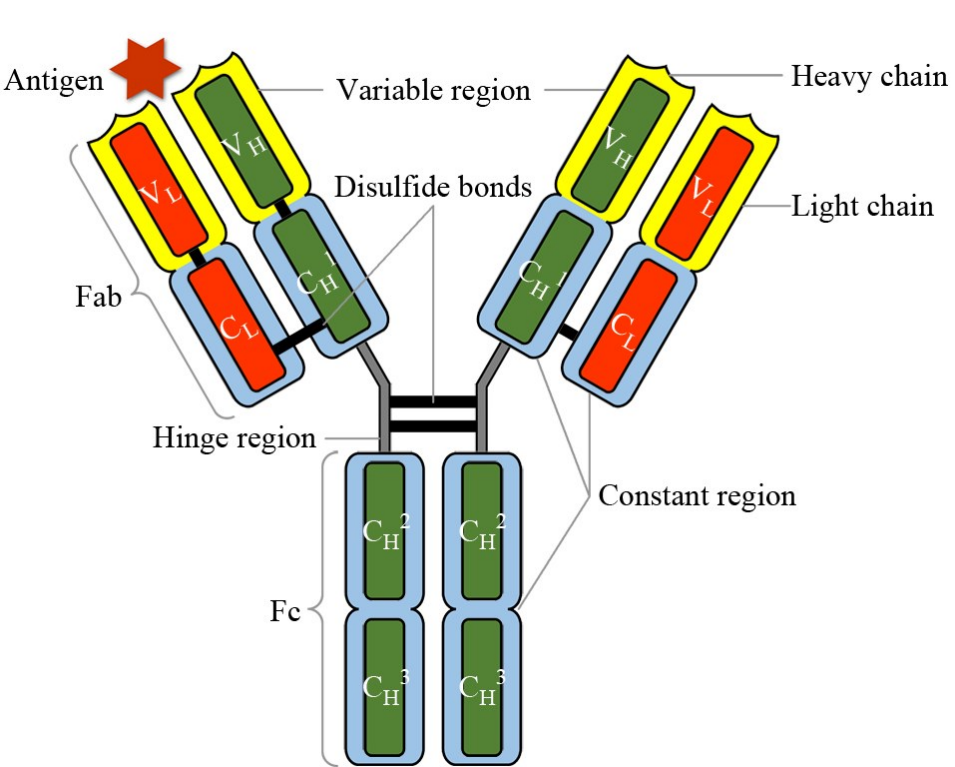
典型抗体IgG的基本结构

鉴于抗体在疾病预防、诊断和治疗中的巨大优势,目前已有超过100个抗体药物被批准上市,特别是单抗药物已经在生物药市场占据了主导地位,全球每年的市场销售收入已超过千亿美元^[6]^。抗体一般来源于动植物体液(如血浆、血清、植物提取液等)或通过细胞工程表达产生,故在原料液中抗体蛋白含量很低,同时有大量DNA、杂蛋白甚至病毒与抗体共存,需要通过一系列分离纯化过程才能获得高纯度产品。尤其是随着上游培养技术的不断发展,单克隆抗体(mAb)的生产能力已经达到g/L级,给下游的纯化工艺带来了巨大压力,导致抗体的纯化成本居高不下,约占抗体生产总成本的50%~80%^[1,7]^。在抗体药物的生产过程中,下游抗体纯化流程一般包括3个步骤(图2), (1)原料液预处理:收集动植物体液或细胞培养液,通过离心或过滤等手段除去细胞碎片;(2)抗体捕获:利用亲和色谱(affinity chromatography, AC,主要是蛋白A亲和色谱)从料液中选择性捕获抗体;(3)抗体精纯:采用色谱等分离技术进一步去除抗体产品和纯化过程中的相关杂质(聚集体、宿主细胞蛋白(HCP)、DNA、病毒、热源等),最终获得高纯度抗体产品^[4,7]^。因此,抗体的分离纯化技术对于抗体药物的生产十分关键。理想的纯化方法应兼备高效、快速、高选择性、低成本等要求,以满足获取高质量抗体产品的目的。

**图 2:**
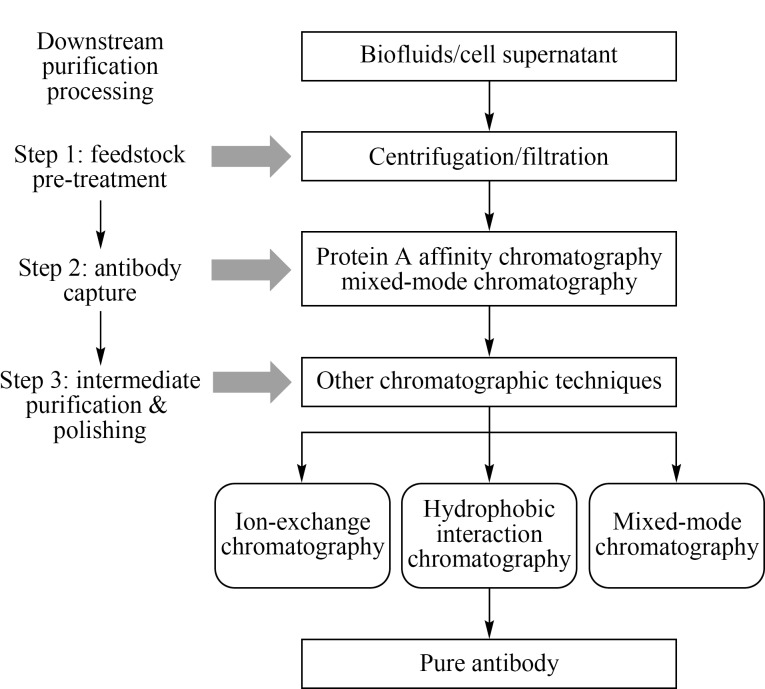
下游抗体纯化流程

抗体的分离纯化方法可分为非色谱法和色谱法两类。非色谱法包括盐析、沉淀、超滤、双水相萃取、磁珠分离以及高效切向流过滤技术等^[1,2,8]^。非色谱法可以有效降低生物料液中的杂质含量,并对抗体进行粗提,但获得的产品纯度较低且纯化技术放大困难,限制了其在抗体分离中的应用。从图2抗体药物的下游纯化过程可以看出,色谱法在抗体分离纯化中占据主导地位,是制备高质量、高纯度抗体的主要手段。用于抗体纯化的色谱技术有亲和色谱、离子交换色谱(ion-exchange chromatography, IEC)、疏水作用色谱(hydrophobic interaction chromatography, HIC)、混合模式色谱(mixed-mode chromatography, MMC)等,各种色谱技术分离机制各异,应用于抗体纯化过程的不同阶段,最终实现复杂原料液中抗体分子的有效提纯。本文将着重介绍各类色谱技术在抗体分离纯化中的应用进展,同时也对近些年出现的新型色谱技术如温敏色谱(temperature-responsive chromatography, TRC)在抗体纯化中的应用研究展开综述,并对抗体色谱纯化技术的发展趋势进行展望。

## 1 亲和色谱

亲和色谱是抗体分离纯化中应用最广、最有效的色谱技术,具有高特异性和高选择性,同时可以减少纯化步骤,缩短纯化时间^[2]^。亲和色谱技术基于偶联在固相基质上的亲和配体与抗体分子之间的特异性识别作用,实现对原料液中抗体蛋白的高效捕获和纯化。亲和色谱具有抗体特异性识别能力的关键在于亲和配体,理想的配体应该与抗体具有高特异性结合力,以达到选择性捕获抗体的目的,同时又能在较温和的条件下与抗体解离,实现抗体的高质量回收。当前用于抗体纯化的亲和配体种类很多,可分为生物特异性配体、合成配体、亲和仿生配体等。

### 1.1 生物特异性配体

生物特异性配体主要是天然蛋白,包括细菌蛋白(如蛋白A、蛋白G和蛋白L)、抗原、凝集素等,它们对抗体分子具有高亲和常数(*K*_a_≈10^5^~10^8^ L/mol)和选择性。因此,基于这些生物特异性配体的亲和色谱一直是亲和纯化抗体的有力工具^[1]^,其中最典型的是蛋白A亲和色谱。蛋白A是金黄色葡萄球菌的细胞壁蛋白,能够与抗体分子的Fc段产生强烈的特异性结合^[9]^,所发展的蛋白A亲和色谱技术是目前规模化分离纯化抗体的首选,可直接从料液中捕获抗体蛋白。蛋白A亲和色谱介质通常在中性pH条件下与抗体结合,并在酸性条件下与其发生解离(pH 3~4),使抗体洗脱。有研究表明,IgG的Fc片段区域中有高度保守的组氨酸残基,与蛋白A中的互补组氨酸残基结合。这些组氨酸残基在低pH下携带正电荷,导致抗体与蛋白A通过静电排斥而被洗脱^[7]^。此外,在洗脱前采用适当的清洗步骤,可有效去除大部分HCP、DNA以及内毒素等杂质,抗体纯度可达98%以上^[4,10]^。除蛋白A外,蛋白G和蛋白L也被用于抗体纯化。蛋白G来源于化脓性链球菌的细胞壁蛋白,与蛋白A和抗体的结合部位相同,结合力弱于蛋白A^[11]^。蛋白L是从消化链球菌中分离出来的蛋白,对于抗体可变区域的*κ*链片段有很强的亲和力^[12]^。3种细菌蛋白的性质如表1所示,它们已实现了重组表达和生产,并用于亲和纯化不同来源的各类抗体亚型。

**表 1:** 用于抗体亲和纯化的3种细菌蛋白的性质^[7]^

Affinity binding protein	Molecular weight/kDa	Binding domains	Ig binding target
Protein A (SpA)	40-60	5 for IgG (most common form)	heavy chain constant region (Fc) of IgG (CH_2_-CH_3_ region)
Protein G (SpG)	40-65	1 to 2 for IgG, 0 to 2 for human	heavy chain constant region (Fc) of IgG (CH_2_-CH_3_ region)
		serum albumin (HSA)	
Protein L (SpL)	76	5 for Ig	Kappa (κ) light chains of Igs (V_L_-κ)

尽管基于细菌蛋白亲和色谱的抗体纯化工艺已得到广泛应用,但该技术存在一些瓶颈问题。例如蛋白A层析介质制备难度大,价格昂贵,增加了抗体生产成本;纯化过程中配体易发生脱落,造成抗体污染;洗脱条件苛刻,酸性洗脱易引起抗体聚集失活;碱性条件下稳定性差,导致层析介质再生性差,缩短了使用寿命。因此,针对蛋白A配体的改造和替代开发一直是抗体纯化研究的重点。例如采用蛋白工程技术对蛋白A进行改造,使其具有更强的耐碱能力,并且提高了洗脱pH^[9]^。利用基因工程对蛋白A的B结构域进行突变,并通过聚合形成四聚体“Z”配体,可以提高蛋白A的耐碱性^[13]^。Honda等^[14]^采用结构设计和组合筛选方法,利用组氨酸扫描文库筛选并开发了D36H突变型蛋白A,实现了在pH 5.0温和条件下对IgG的高效洗脱。Katakura等^[15]^以突变的热敏蛋白A为配体,与IgG在低温下(5 ℃)结合,而在高温下(40 ℃)解离释放,实现了中性条件下(pH 8.0)抗体的洗脱。此外,通过对蛋白A分子中抗体识别区域进行聚合(如四聚体结构域Z),可以提高蛋白A对抗体的结合容量;通过将蛋白A分子以一定的空间取向固载在基质表面,能够提高对抗体的结合效率^[16]^。蛋白A的工程改造一定程度上提高了其在抗体纯化过程中的稳定性、结合效率和结合容量,但蛋白配体的一些固有缺点如制备成本高、易脱落等仍然无法避免。

### 1.2 合成配体

合成配体是由人工合成的一类与抗体有特异性结合能力的有机或有机/无机化合物。与天然蛋白配体相比,合成配体的可设计性更强,耐用性更高,合成成本更低。合成配体类型包括亲硫配体、金属螯合配体、染料配体、硼酸亲和配体、超分子配体等。

亲硫色谱(thiophilic chromatography, TC),也称嗜硫色谱,通常以二乙烯基砜与2-巯基乙醇反应为基础,在琼脂糖表面形成含有两个硫原子的线性配体(-CH_2_CH_2_SO_2_CH_2_CH_2_SCH_2_CH_2_OH)^[17]^。亲硫配体在高盐条件下表现出对抗体的特异性亲和力,通过降低盐浓度实现洗脱。亲硫色谱已广泛应用于纯化不同类型的抗体,包括哺乳动物IgG,蛋黄IgY,小鼠IgE、Fab片段和大肠杆菌的单链抗体片段(scFvs)等^[2,17,18]^。高盐环境对抗体活性不利,开发不依赖盐的新型亲硫配体可以改善这一情况。Qian等^[19,20]^合成了两种巯基杂环配体(2-巯基烟酸和2-巯基-1-甲基咪唑)功能化的磁珠,可在较低盐浓度下实现人血清中IgG的洗脱,抗体活性高于98%。

固定化金属亲和色谱(immobilized metal affinity chromatography, IMAC)以金属螯合配体为亲和基团,利用蛋白质暴露的氨基酸残基(组氨酸、色氨酸、天冬氨酸等)与介质上的金属离子(Cu^2+^、Ni^2+^、Co^2+^等)之间的配位作用分离蛋白质。IMAC技术已实现对不同来源、不同亚型抗体的纯化^[3,5,21]^,特别是对于通过大肠杆菌表达的带有组氨酸标签的单抗(HiP4-tag mAb)^[22]^或单域抗体(sdAb)包涵体的分离纯化^[23]^, IMAC技术展现出了独特的优势。IMAC分离条件比较温和,在中性条件下结合,通过改变pH或加入竞争试剂(咪唑)洗脱蛋白,但IMAC对抗体的亲和特异性较低,还存在金属离子泄露的风险。

染料配体亲和色谱(dye-ligand affinity chromatography, DAC)以染料分子为配体,通过模仿底物、辅助因子或结合剂的结构,与许多蛋白质和酶的活性位点产生特异性相互作用,已用于多种蛋白质的分离和纯化^[24,25]^。染料配体价格低廉,易于通过反应基团偶联到各种基质表面,并且耐化学和生物降解。一些三嗪类染料分子如活性绿HE 4BD、活性红120、活性绿5以及汽巴蓝3G-A在人和兔IgG及其亚型的分离纯化中表现出良好的性能^[24⇓-26]^。Huseynli等^[27]^采用活性红120和活性绿HE 4BD染料对聚甲基丙烯酸2-羟乙基(PHEMA)微球进行了功能化,并将其包埋在超大孔冷冻凝胶中制备了复合低温凝胶片,可以在较低背压下使用高流速对人IgG及其亚类进行亲和分离,有效提高了抗体的纯化效率,同时冷冻凝胶高比表面积增大了材料对IgG的吸附容量。

硼酸亲和色谱(boronate affinity chromatography, BAC)是基于硼酸与顺式二羟基在碱性水溶液中形成稳定的环酯,选择性捕获含有糖分子基团的蛋白质。硼酸酯具有可逆性,在酸性条件下发生解离,实现目标蛋白洗脱^[28]^。IgG是一种糖蛋白,在Fc区存在聚糖基团,可利用硼亲和原理进行纯化^[29,30]^。硼酸亲和色谱具有成本低、稳定性好和洗脱快等优点,但样品中共存的糖蛋白会与抗体在硼酸亲和介质表面发生竞争结合,降低了对抗体的选择性。最近,硼酸亲和色谱技术作为检测糖基化水平的工具,实现了对mAb糖基化的准确测定^[31]^,该研究为实时监控生物过程中抗体的糖基化动态提供了新方法。

超分子大环化合物因具有特殊的主客体相互作用而受到广泛关注。Kim等^[32]^利用葫芦脲[7](CB[7])与金刚烷胺(AdA)之间强烈的主客体识别性质,合成了CB[7]接枝的琼脂糖亲和介质,并通过基因工程和酶修饰技术,对细胞表达的mAb末端用AdA进行标记。抗体通过末端AdA与CB[7]配体之间的特异性主客体识别作用被捕获到亲和介质表面,再使用与CB[7]主客体作用更强的*N*-(1-金刚烷基)乙二胺(Ad-EDA)将抗体置换洗脱下来。与蛋白A亲和纯化结果相比,该CB[7]亲和介质具有更高的抗体回收率,而且杂质含量比蛋白A介质降低了2.4倍。此外,CB[7]介质经过简单的高盐洗涤除去Ad-EDA后,还可实现多次循环使用。超分子配体具有灵活的结构设计、强烈的主客体亲和作用以及高度的稳定性和耐用性,有望在亲和纯化抗体方面得到更多的关注。

### 1.3 亲和仿生配体

亲和仿生配体亦属于合成配体,但该类配体是针对抗体开展理性设计,采用不同的方式建立仿生配基库,通过高通量筛选技术发现潜在的特异性配基,再以这些配基制备亲和仿生介质,与目标抗体进行结合实验,最终筛选得到对抗体具有高特异性结合能力的亲和仿生配体^[33]^。因此亲和仿生配体与抗体的亲和特异性比普通的合成配体高得多。随着组合化学、高通量筛选技术、蛋白质晶体结构解析以及计算机分子模拟的快速发展,越来越多的亲和仿生配体被发现并应用于抗体分离纯化中。

亲和仿生配体主要有化学合成仿生配体和短肽仿生配体两类。化学合成仿生配体采用从头设计策略,以三嗪结构或多组分反应合成(Ugi反应)为基础,将计算机模拟、组合化学以及层析筛选等方法相结合,分析抗体和天然配体间的结合模式,确定天然配体的关键残基,并以此作为模板合成亲和仿生配体^[33]^。例如结合分子模拟和组合化学技术开发的基于三嗪骨架结构的合成仿生配体Ligand 22/8(蛋白A为模板)、Ligand 8/7(蛋白L为模板),对抗体以及Fab和scFV片段具有良好的分离纯化性能^[33,34]^。最近,Safary等^[35]^在琼脂糖表面合成了一系列三嗪双取代基亲和仿生配体,并以人和兔血清中IgG为目标蛋白,对仿生配体的纯化性能进行了层析筛选。结果表明,含有两个3-氨基苯酚取代基团的仿生配体对IgG具有最佳的分离选择性、蛋白回收率和纯度。Ugi反应合成是将活化介质、羧酸化合物、伯胺以及异腈结构同时加入反应体系中,通过一步化学合成法得到仿生配体^[33]^。Khoury等^[36]^模拟蛋白G结合域Ⅲ中的关键氨基酸残基,采用四组分Ugi反应合成了仿生配体A2C711,成功实现了IgG和Fac片段的高效纯化,纯度分别为99%和93%。Ugi反应策略增加了配体结构的多样性,节约了试剂用量,缩短了反应时间,有利于亲和介质的放大生产。

短肽仿生配体是含有2~9个特定氨基酸序列的多肽化合物,有线性肽、环状肽和多聚肽等多种形式,与抗体分子之间存在特异性亲和作用^[33,37]^。短肽仿生配体的开发一般经过理性设计、短肽库构建、配体筛选与合成等步骤,最后将潜在的高亲和仿生多肽配体接枝在介质表面,验证抗体分离纯化性能。短肽的理性设计可基于抗体的整体三维结构或活性部位信息开展,找到关键氨基酸残基,并据此构建合理的仿生短肽库,缩小筛选范围;配体筛选可选用组合化学策略、mRNA展示技术、噬菌体展示技术或计算机辅助技术等,从短肽库中筛选出对抗体特定靶点部位有高亲和作用力的潜在短肽,并确定其氨基酸序列^[33,37]^。短肽仿生配体是抗体亲和纯化领域的研究热点,除了文献[16,37]中报道的短肽配体外,近些年又涌现出了一系列新的短肽配体。Lin课题组^[38]^在2019年以IgG分子Fc片段中与配体结合的关键氨基酸残基为基础,选择4个残基随机组合形成四肽库,并通过分子对接筛选出LibDock评分最高的新型四肽配体Ac-FYHE。将该配体偶联到琼脂糖凝胶表面,分别实现了细胞培养液和人血清中mAb和IgG的高选择性纯化,一步分离纯度和回收率均高于90%。2021年,该课题组^[39]^采用相似的策略又从480个四肽的肽库中筛选出4个四肽配体(Ac-FYKH、Ac-YEHF、Ac-YFLH和Ac-FYHI),并进一步将其接枝在琼脂糖凝胶上,通过色谱分离从中筛选出纯化性能最佳的亲和树脂^[40]^。4个四肽配体中,Ac-YEHF对抗体的结合能力最佳,分离效果最好。除了开发新的短肽仿生配体外,深入研究已有短肽配体与抗体或其他杂质的结合情况^[41]^,或将其接枝在新的基质中提高抗体纯化性能^[42]^,可为短肽亲和分离技术的进一步发展和完善提供有益探索。总体来说,亲和仿生色谱(affinity biomimetic chromatography, ABC)作为一种新型生物分离技术,与蛋白A配体相比,短肽亲和配体对抗体蛋白亲和性强,且不易被降解,具有良好的选择性和稳定性,价格低廉,耐原位清洗,在抗体纯化中表现出良好的应用前景。

## 2 离子交换色谱

离子交换色谱是一种重要的生物大分子分离技术,具有处理量大、分离条件温和、操作重复性高等优势,能有效去除与产品相关的杂质和污染物,在下游工艺中主要用于抗体的精纯。离子交换色谱分为阴离子交换和阳离子交换2种模式,阴离子交换树脂(AEX)能够去除内毒素、病毒、HCP和DNA,而阳离子交换树脂(CEX)则用于分离电荷变体、蛋白聚集体、相对分子质量较低的降解产物以及HCP^[8,43]^。IEC的性能受分离过程中多个参数的共同影响,包括蛋白质性质、流动相条件以及固定相特性(如基质类型和孔隙度、配体密度等)^[44]^。因此,深入研究参数之间的复杂相互关系及其对抗体纯化效果的影响,可以为抗体分离工艺的设计、优化以及规模扩大提供有用信息。Frech等^[43]^在阴离子交换模式下,构建了用于考察配体密度对蛋白质保留影响的模型,并研究了13种配体密度的阴离子交换树脂对白蛋白和mAb保留行为的影响。结果证实模型模拟结果与实验结果一致,能够描述配体密度与蛋白质保留之间的依赖关系。Hubbuch等^[45]^建立了一种多尺度的预测模型,包括同源性建模、定量结构-性质关系(QSPR)以及从单抗氨基酸序列到阳离子交换机制的机械色谱模型。该多尺度模型可以基于抗体的氨基酸序列,预测整个CEX色谱分离结果而无需初始实验。上述理论模型可为加速开发离子交换纯化工艺,并深入理解离子交换过程中各种因素的相互作用机制提供指导。

## 3 疏水作用色谱

疏水作用色谱是利用盐-水体系中样品分子的疏水基团和介质的疏水配体之间疏水作用力的差异而进行分离的技术。抗体分子表面暴露的非极性氨基酸侧链聚集形成疏水区域,可与疏水介质产生相互作用而实现分离^[46]^。大多数情况下,HIC在高盐浓度下上样,低盐浓度下洗脱,具有分离条件温和,蛋白质回收率高、蛋白活性高的特点,而且操作简单、处理量大,通常用于抗体的精纯^[46,47]^。Jungbauer等^[47]^利用HIC技术对抗肿瘤坏死因子-*α*(TNF-*α*)抗体进行精纯,抗体纯度可达99.9%。除了抗体精纯外,HIC还可用于抗体-药物偶联物(ADC)的纯化与表征。Müller等^[48]^选择5-异硫氰酸荧光素代替有毒的细胞药物,与抗体偶联构建了ADC模型,建立了基于HIC机制的分析与纯化方法,成功实现了药物与抗体偶联比例的测定,并对偶联物进行了提纯。HIC在抗体结构和纯度表征方面(如抗体翻译后修饰、抗体与药物复合比例测定、抗体疏水性以及双抗异源二聚体分析等)也有广泛的应用^[49]^,还能与其他技术联用,对抗体开展深入精准的分析^[50]^。

## 4 混合模式色谱

混合模式色谱是在固定相上键合多种功能基团,利用固定相和溶质分子之间的多重作用机制,实现复杂样品精准分离的技术^[51]^。与单一作用模式色谱相比,MMC通过设计合理的配体结构,可以将各种分离机制(离子交换、疏水作用、氢键作用)进行灵活组合,增强了固定相对溶质分子的选择性和负载容量,同时拓宽了溶质保留的盐浓度范围,减少了对原料液的预处理,提高了分离效率^[52]^。因此,MMC色谱技术在抗体分离纯化中受到了极大关注。

### 4.1 疏水电荷诱导色谱

疏水/离子交换模式是MMC纯化抗体时最常用的分离机制,其中疏水电荷诱导色谱(hydrophobic charge-induction chromatography, HCIC)利用氮杂环配体在不同pH下疏水和电荷性质的不同,通过控制pH变化实现抗体的捕获和洗脱。经典的HCIC配体为4-巯基吡啶(MEP, p*K*_a_ 4.8),对应的商品化介质称为MEP HyperCel。该介质以纤维素为基质,MEP为配体,其结构中含有硫原子、吡啶杂环和疏水链,在中性条件下可以对抗体产生特异性吸附,具有不依赖盐的特性。当调节pH至酸性(pH<p*K*_a_),使吡啶环带正电荷,与同样带正电荷的抗体产生静电排斥作用,可实现抗体的洗脱^[53]^。基于MEP HyperCel树脂的HCIC技术已成功实现了哺乳动物乳液、细胞培养液、细菌提取液等中抗体的分离纯化,部分抗体的纯化效果与蛋白A相当^[53]^。Lin等^[54,55]^通过分子对接模拟和等温滴定量热法研究了MEP配体与IgG分子的作用力类型和结合位点,发现在中性pH下MEP可与IgG Fc片段的TYR319和LEU309附近位点稳定结合,且疏水相互作用是主要驱动力,并伴有范德华力和氢键作用。而在酸性条件下,MEP与Fc片段产生静电斥力,同时疏水作用减弱和氢键消失,使MEP快速与Fc脱离。此外,MEP配体中的硫原子也会为抗体结合提供额外的亲硫作用力^[56]^。除了MEP配体外,其他一些具有HCIC特性的配体也有报道,如表2所示。这些配体的共同特点是均含有疏水基团和氮杂环结构,对抗体的保留机理与MEP相似。这些配体对抗体的保留规律及分离性能均已得到了深入研究^[53,57⇓⇓⇓⇓⇓⇓-64]^,为利用混合模式分离纯化抗体提供了多种选择。除了设计开发新的HCIC配体外,影响HCIC对抗体分离纯化效果的因素还有基质类型、配体密度、配体接枝方法、流动相添加剂等,需要通过实验进行考察和优化。

**表 2:** 文献报道的HCIC配体及其蛋白质纯化性能

Ligand	Structure	pK_a_	Target	Feedstock	Purity/%	Recovery/%	Ref.
4-Mercaptoethyl-pyridine (MEP)	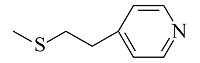	4.8	IgG	bovine colostrums	93.9	91.5	[57]
2-Mercapto-1-methylimidazole (MMI)	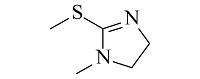	5.3	IgY (ΔFc)	goose blood	98.6	85.0	[58]
5-Aminobenzimidazol (ABI)	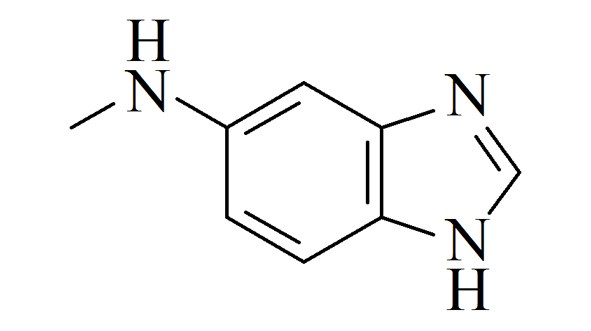	6.5	monoclonal IgM	cell culture supernatant	99	80.8	[59]
2-Mercaptobenzimidazole (MBI)	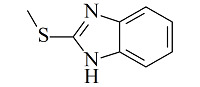	4.1	IgG	IgG/BSA mixture	~90	96	[60]
Histamine	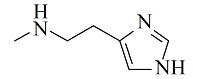	6.04	lysozyme	protein solution	N.A.	N.A.	[61]
5-Aminoindole (AI)	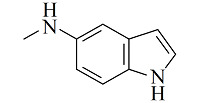	3.9	lysozyme	protein solution	~90	~90	[62]
2-Mercaptoimidazole (MI)	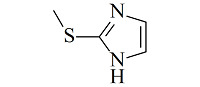	6.5	IgG	porcine plasma	89.4	N.A.	[63]
Benzotriazole-5-carboxylic (BTA)	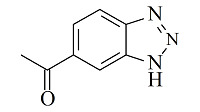	N.A.	mAb	cell culture supernatant	89.7	95.1	[64]

HCIC: hydrophobic charge-induction chromatography; N.A.: not available.

HCIC技术具有分离条件温和、耐盐性强、吸附容量大等优点,同时配体的制备成本低、化学稳定性高,可显著降低抗体的纯化成本,有望成为蛋白A亲和色谱的替代品。然而HCIC配体的选择性仍低于蛋白A,而且抗体的洗脱亦需在较强的酸性条件(pH<4)下进行,不利于保持抗体的生物活性。Lin等^[65]^提出多模态电荷诱导色谱,将HCIC配体5-氨基苯并咪唑与色氨酸连接,设计了新的MMC配体W-ABI,并将其接枝在琼脂糖凝胶上,研究对抗体的吸附选择性。该配体与抗体Fc结构域产生多模式协同相互作用,增强了二者间的结合力,对抗体的纯化效果比商品化MEP HyperCel更好,而且抗体在pH 5.0条件下可以实现高效洗脱,避免了强酸环境对其结构和活性的影响。该课题组还将短肽仿生配体与HCIC配体结合,设计合成了新型杂化仿生配体FYE-ABI,同样增强了对抗体的捕获选择性,洗脱条件也比较温和(pH 4.5),对人血清中IgG的一步纯化效果与蛋白A层析相当^[66]^。将多种作用模式的配体进行有效融合,设计开发新型亲和-混合模式分离技术,是提高抗体分离选择性、改善洗脱条件的有效途径。

### 4.2 离子交换/疏水混合模式色谱

离子交换/疏水混合模式色谱(ion exchange/hydrophobic interaction mixed-mode chromatography, IEC/HIC MMC)同样利用离子交换和疏水基团与抗体产生相互作用。HCIC中离子交换机制并非用于抗体的结合,而是促进抗体的洗脱,但在IEC/HIC MMC中固定相对抗体的捕获是在疏水和静电共同作用下完成的^[67]^,其中以疏水作用为主导。IEC/HIC MMC的配体类型有含氮基团的烷基型化合物,如Pall公司的HEA HyperCel;还有含氮或羧基基团的苯基型化合物,如GE公司的Capto MMC和Capto Adhere以及Bio-Rad公司的Nuvia cPrime介质。HEA HyperCel和Capto Adhere含有季铵基团,是强阴离子交换/疏水混合模式介质,Capto MMC和Nuvia cPrime含有羧基,为弱阳离子交换/疏水混合模式介质。季铵或羧基基团提供离子交换作用力,苯环和烷基链提供疏水作用力,羟基提供氢键作用^[68]^。IEC/HIC MMC介质已成功用于抗体的纯化^[69⇓-71]^。例如HEA HyperCel可以直接捕获中国仓鼠卵巢细胞(CHO)培养液中的mAb,然后再经过Capto MMC的中级纯化和阴离子交换膜精制后,最终获得纯度超过99.9%的抗体产品,每一步纯化的回收率为94%~99%,杂质水平低于规定值^[72]^。最近,IEC/HIC MMC在双特异性抗体(bsAb)及其杂质分离和去除中表现出良好的潜力。使用Capto MMC ImpRes树脂,在最佳条件下可以有效除去Wuxibody中伴随的轻链缺失副产物以及聚集体^[73]^;而Capto MMC和Capto Adhere则可分离双特异性抗体与杂质,其中Capto Adhere因季铵基团提供了更强的静电排斥作用,能够更好地洗脱疏水性较强的bsAb,回收率比Capto MMC更高^[74]^。

离子液体是一类由有机阳离子和无机/有机阴离子组成的液态盐类物质,因其独特的结构设计性,在生物分子及手性物质色谱分离中得到广泛应用^[75,76]^。离子液体中包含阴阳离子基团和疏水烷基链,因此将离子液体固定在载体表面后,具备了IEC/HIC MMC分离功能。Capela等^[77]^在硅胶基质表面固定了3种功能化离子液体:1-甲基-3-丙基咪唑基([C_3_mim]Cl)、三丁基丙基铵([N_3444_]Cl)和三辛基丙基铵([N_3888_]Cl),并首次应用于IgG的分离纯化。3种离子液体固定相中,[N_3444_]Cl和[N_3888_]Cl两种固定相能够通过静电和疏水作用,在中性条件下选择性保留IgG;[C_3_mim]Cl固定相在酸性溶液中仅保留杂质白蛋白,而IgG则直接流出色谱柱,实现分离纯化。基于离子液体配基与蛋白质相互作用的差异,研究者建立了两种抗体纯化的新方法,成功实现了动物血清和细胞培养液中IgG的分离提纯,回收率和纯度良好。该研究表明,利用离子液体独特的可设计性,调控离子交换和疏水基团的类型及比例,有望开发一系列新型离子交换/疏水混合模式固定相并应用于抗体纯化。

### 4.3 羟基磷灰石色谱

离子交换/配位作用模式是另一类MMC分离机制,其典型代表为羟基磷灰石色谱(hydroxyapatite chromatography, HAC)。羟基磷灰石由磷酸钙(Ca_10_(PO_4_)_6_(OH)_2_)晶体构成,晶体中的磷酸基团(P-位)通过阳离子交换与蛋白质中的胺或其他带正电的氨基酸残基产生静电相互作用,而表面的钙离子(C-位)则通过金属亲和机制与蛋白质中的羧基簇或磷酸基结合^[5]^。蛋白质在HAC固定相中通过磷酸盐或NaCl盐梯度进行洗脱。HAC在抗体纯化中主要用于抗体精制步骤,除去残留的聚集体、HCP、DNA等杂质^[8,78]^,有时也可替代蛋白A,用于IgA和IgM的纯化^[67]^,还可以用于bsAb的纯化^[74]^。羟基磷灰石作为色谱填料时,存在柱容量低、柱压高、流速低和寿命短等缺点,限制了其在抗体纯化中的应用。而且羟基磷灰石耐酸性差,磷酸钙会发生溶解,用氟原子代替羟基,使用氟磷灰石可以提高介质在酸性环境中的稳定性^[1,8]^。

## 5 温敏色谱

温敏色谱是以温度响应性聚合物修饰的介质为固定相,利用温敏聚合物亲疏水性随外界温度变化而变化的现象,通过调节柱温调控固定相与溶质分子之间的亲疏水作用力,从而实现组分分离的一种新型色谱方法^[79]^。TRC中最常见的温敏聚合物为聚*N*-异丙基丙烯酰胺(PNIPAAm),其在水中的相转变温度(LCST)约为32 ℃。当温度低于LCST时,PNIPAAm与水分子之间形成氢键使其处于拉伸状态,具有亲水性,而当温度高于LCST时,氢键作用变弱导致聚合物链收缩,疏水性增强^[80]^。因此,在TRC中调节色谱柱温度即可改变固定相的亲疏水性质,从而影响溶质的保留行为。TRC中仅以水作为流动相,避免使用有机溶剂和高含量盐,保护了分析物的生物活性,特别适合生物大分子的分离^[79,81]^,在抗体纯化领域也展现了一定的应用价值。Nagase等^[82]^采用原子转移自由基聚合(ATRP)技术在硅胶表面分别修饰了3种三元温敏共聚物:聚NIPAAm-co-2-丙烯酰胺-2-甲基丙磺酸(AMPS)-co-*n*-苯基丙烯酰胺(PhAAm)、聚NIPAAm-co-AMPS-co-*n*-甲基丙烯酸丁酯(BMA)和聚NIPAAm-co-AMPS-co-叔丁基丙烯酰胺(tBAAm)。3种温敏共聚物含有相同的温敏响应基团NIPAAm和强阳离子交换基团SO_3_^-^以及不同的疏水基团。40 ℃下,温敏聚合物收缩,使阳离子交换基团聚集在硅胶表面,提高了电荷密度,有利于利妥昔单抗通过静电作用保留在固定相表面,同时,高温下处于疏水状态的聚合物链也能为抗体保留提供疏水作用力。当温度降至10 ℃时,聚合物链舒展导致电荷密度下降,削弱了与抗体之间的静电作用力,实现了抗体洗脱。3种温敏固定相中,P(NIPAAm-co-AMPS-co-BMA)分离抗体的效果最好,可以实现CHO细胞培养液中利妥昔单抗的分离纯化。此外,通过改变温度,调控聚合物的疏水和静电作用平衡,还可以实现多种单抗的分离,这是蛋白A亲和色谱无法实现的。另一项工作通过表面引发自由基聚合法在硅胶表面接枝了聚NIPAAm-co-BMA-co-丙烯酸(AAC)-co-*N*,*N*'-亚甲基双丙烯酰胺(Bis)交联温敏共聚物,并将该温敏固定相用于mAb的纯化。通过选择不同的盐种类,该固定相分别在25 ℃和40 ℃下成功纯化了利妥昔单抗和曲妥珠单抗^[83]^。温敏色谱纯化抗体条件温和,可有效避免严苛的洗脱条件引起的抗体失活,而且与生物亲和层析介质相比,温敏聚合物介质的制备成本更低,有利于降低抗体纯化成本。

尽管温敏色谱在抗体纯化领域表现出了潜在的竞争力,但温敏聚合物仅通过疏水作用与抗体结合,缺乏特异性,导致抗体纯度降低。将对抗体有特异性亲和作用的配体与温敏聚合物结合,开发温敏亲和色谱固定相,能提高对抗体分离的选择性。我们课题组^[84]^开发了高容量温敏亲和色谱固定相。先通过可逆加成-断裂链转移聚合技术(RAFT)制备了温敏聚合物PNIPAAm,再将其末端与MEP共价偶联,同时选择树枝状聚酰胺-胺(PAMAM)为间隔臂,提高硅胶表面温敏聚合物与亲和配体的接枝密度,从而提高抗体的吸附容量。该温敏亲和固定相在40 ℃下能同时保留牛血清白蛋白(BSA)和*γ*-球蛋白,在5 ℃下即可将*γ*-球蛋白洗脱下来,而无需改变流动相酸度。MEP基团与抗体分子之间存在特异性亲和作用,故抗体与固定相的结合强度高于BSA。因此温度降低后,BSA与固定相之间的疏水作用力消失,率先洗脱下来,*γ*-球蛋白则晚于BSA,从而实现二者的分离。最近,我们课题组^[85]^进一步以嵌段温敏共聚物聚[*N*-异丙基丙烯酰胺-*b*-4-乙烯基吡啶](P[NIPAM-*b*-4VP])为间隔臂,MEP为亲和配体,制备了一种嵌段共聚温敏亲和色谱固定相SiO_2_-P[NIPAM-*b*-4VP]-MEP,并对抗体进行分离纯化。该温敏亲和固定相进一步提高了对抗体保留的选择性,BSA则在该固定相表面无法保留。同样地,抗体仅通过改变温度和适当调节流动相离子强度,即可实现完全洗脱^[85]^。温敏亲和色谱结合了温敏聚合物的温度响应特性和亲和配体的高选择性,分离条件温和、介质制备成本低,有望彻底解决蛋白A亲和色谱酸性洗脱易使抗体蛋白变性失活的问题,成为抗体纯化领域发展的新方向。

## 6 结论与展望

随着抗体药物市场规模的不断扩大,分离纯化环节对抗体大规模生产的制约效应日益凸显。时至今日,色谱分离技术仍然是抗体分离纯化最有效的手段,也是决定抗体生产成本的关键因素。目前蛋白A亲和色谱承担了约2/3的抗体纯化任务,主要从上游原料液中高选择性捕获抗体。其他色谱分离技术如离子交换色谱、疏水作用色谱、羟基磷灰石色谱等则用于抗体的后续精纯。亲和仿生色谱和混合模式色谱在抗体纯化中受到了较多关注,近些年在组合化学、计算机模拟等技术辅助下,开发出了许多新型的仿生配体及混合模式固定相,在抗体纯化中表现出良好的性能。温敏色谱因温敏聚合物的特殊性质,仅通过改变温度即可实现抗体的捕获与洗脱,最大程度克服了蛋白A亲和色谱的酸性洗脱问题,有望为抗体纯化领域带来突破性变革。另外,超分子配体通过强烈的主客体相互作用,显著提高了对特定标签抗体的特异性识别能力,为开发超分子亲和色谱提供了新的可能。然而迄今为止,蛋白A亲和色谱在抗体纯化领域的地位仍无法替代,其他色谱技术如混合模式色谱、温敏色谱、超分子亲和色谱等在抗体捕获的选择性、相互作用机制、分离条件优化、杂质控制等方面的研究仍显不足。因此,今后色谱技术应从以下方面开展深入研究,不断满足抗体分离纯化的需求:①进一步发展具有更高特异性和更温和分离条件的新型亲和仿生配体、混合模式配体和超分子配体;②系统研究温敏聚合物结构组成与抗体纯化性能之间的规律;③融合各种分离机制,发展选择性好、洗脱条件温和的多模态色谱分离方法。相信随着研究的逐步深入和新技术新方法的不断涌现,更加简单、高效、高选择性、高质量和低成本的抗体纯化技术将为抗体药物的生产和广泛应用提供重要的技术支撑。

## References

[1] Roque A C A, Silva C S O, Taipa M A. J Chromatogr A, 2007, 1160(1/2): 44 17618635 10.1016/j.chroma.2007.05.109

[2] Zhai D G, Chen L, Qian P. Biological Chemical Engineering, 2018, 4(3): 145

[3] Arora S, Saxena V, Ayyar B V. Methods, 2017, 116: 64 10.1016/j.ymeth.2016.12.010 28012937

[4] Lu H L, Lin D Q, Yao S J. CIESC Journal, 2018, 69(1): 341

[5] Ayyar B V, Arora S, Murphy C , et al. Methods, 2012, 56(2): 116 22033471 10.1016/j.ymeth.2011.10.007

[6] Zhang H, Zhang J Y, Wu H R , et al. Membrane Science and Technology, 2023, 43(5): 150

[7] Chahar D S, Selvan R, Pisal S S. Biologicals, 2020, 63: 1 31558429 10.1016/j.biologicals.2019.09.007

[8] Gagnon P. J Chromatogr A, 2012, 1221: 57 22071423 10.1016/j.chroma.2011.10.034

[9] Zarrineh M, Mashhadi I S, Farhadpour M , et al. Anal Biochem, 2020, 609: 113909 32827466 10.1016/j.ab.2020.113909

[10] Kangwa M, Yelemane V, Ponnurangam A , et al. Protein Expres Purif, 2019, 155: 27 10.1016/j.pep.2018.11.003 30445097

[11] Bailey L J, Sheehy K M, Hoey R J , et al. J Immunol Methods, 2014, 415: 24 25450256 10.1016/j.jim.2014.10.003 PMC4257880

[12] Paloni M, Cavallotti C. ACS Omega, 2017, 2(10): 6464 31457247 10.1021/acsomega.7b01123 PMC6645367

[13] von Roman M F, Berensmeier S. J Chromatogr A, 2014, 1347: 80 24819019 10.1016/j.chroma.2014.04.063

[14] Tsukamoto M, Watanabe H, Ooishi A , et al. J Biol Eng, 2014, 8: 15 25057290 10.1186/1754-1611-8-15 PMC4107488

[15] Koguma I, Yamashita S, Sato S , et al. J Chromatogr A, 2013, 1305: 149 23891370 10.1016/j.chroma.2013.07.015

[16] Shi Q H, Sun Y. Chin J Chem Eng, 2021, 30: 194

[17] Boschetti E. J Biochem Biophys Methods, 2001, 49(1-3): 361 11694289 10.1016/s0165-022x(01)00208-1

[18] Qian H, Li C J, Lin Z Y , et al. Colloid Surface B, 2010, 75(1): 342 10.1016/j.colsurfb.2009.09.007 19783128

[19] Lin Z Y, Zhang Y X, Li C J , et al. J Immunol Methods, 2021, 489: 112914 33197470 10.1016/j.jim.2020.112914

[20] Qian H. Colloid Surface B, 2013, 108: 72

[21] Cheung R C F, Wong J H, Ng T B. Appl Microbiol Biotechnol, 2012, 96(6): 1411 23099912 10.1007/s00253-012-4507-0

[22] Ino Y, Yamaoka Y, Tanaka K , et al. Proteomics, 2023, 23(11): 2200334 10.1002/pmic.202200334 36807525

[23] Maggi M, Scotti C. Data Brief, 2017, 12: 132 28413818 10.1016/j.dib.2017.03.039 PMC5384857

[24] Yavuz H, Akgol S, Say R , et al. Int J Biol Macromol, 2006, 39(4/5): 303 16764922 10.1016/j.ijbiomac.2006.04.009

[25] Wongchuphan R, Tey B T, Tan W S, et al. Biochem Eng J, 2009, 45(3): 232

[26] Gondim D R, Lima L P, de Souza M C M , et al. Adsorpt Sci Technol, 2012, 30(8/9): 701

[27] Huseynli S, Baydemir G, Sari E, et al. Mater Sci Eng C, 2015, 46: 77 10.1016/j.msec.2014.10.007 25491962

[28] Li D J, Chen Y, Liu Z. Chem Soc Rev, 2015, 44(22): 8097 26377373 10.1039/c5cs00013k

[29] dos Santos R, Rosa S A S L, Aires-Barros M R , et al. J Chromatogr A, 2014, 1355: 115 24947887 10.1016/j.chroma.2014.06.001

[30] Rosa S A S L, Wagner A, da Silva C L, et al. Biotechnol J, 2019, 14(10): 1800586 10.1002/biot.201800586 31305007

[31] Lhota G, Sissolak B, Striedner G , et al. Biotechnol Progress, 2021, 37(3): e3124 10.1002/btpr.3124 PMC8365726 33428326

[32] An J, Kim S, Shrinidhi A , et al. Nat Biomed Eng, 2020, 4(11): 1044 32690883 10.1038/s41551-020-0589-7

[33] Lu H L, Lin D Q, Yao S J. CIESC Journal, 2016, 67(9): 3523

[34] Xue A Y, Fan S J. Biomolecules, 2022, 12(6): 821 35740946 10.3390/biom12060821 PMC9221399

[35] Farzi-Khajeh H, Toraby S, Akbarzadeh-Khiavi M , et al. J Chromatogr A, 2022, 1684: 463559 36257172 10.1016/j.chroma.2022.463559

[36] El Khoury G, Lowe C R. J Mol Recognit, 2013, 26(4): 190 23456743 10.1002/jmr.2265

[37] Fang Y M, Lin D Q, Yao S J. J Chromatogr A, 2018, 1571: 1 30097342 10.1016/j.chroma.2018.07.082

[38] Fang Y M, Chen S G, Lin D Q , et al. J Chromatogr A, 2019, 1604: 460474 31493850 10.1016/j.chroma.2019.460474

[39] Fang M Y, Lin D Q, Yao S J. Biochem Eng J, 2021, 176: 108213

[40] Fang Y M, Zhang Q L, Lin D Q , et al. J Chromatogr B, 2022, 1208: 123407 10.1016/j.jchromb.2022.123407 35964489

[41] Huang H T, Dong X Y, Sun Y , et al. J Chromatogr A, 2023, 1707: 464305 37607431 10.1016/j.chroma.2023.464305

[42] Wang X Y, Xia D H, Han H , et al. Anal Chim Acta, 2018, 1017: 57 29534796 10.1016/j.aca.2018.02.012

[43] Sanchez-Reyes G, Graalfs H, Hafner M, et al. J Sep Sci, 2021, 44(4): 805 33285038 10.1002/jssc.202001077

[44] Hardin A M, Harinarayan C, Malmquist G , et al. J Chromatogr A, 2009, 1216(20): 4366 19344903 10.1016/j.chroma.2008.08.047 PMC2997668

[45] Saleh D, Hess R, Ahlers-Hesse M , et al. Biotechnol Bioeng, 2023, 120(1): 125 36226467 10.1002/bit.28258

[46] Shen H, Lin Q Q, Yi Y , et al. Pharmaceutical Biotechnology, 2012, 19(5): 401

[47] Bresolin I R A P, Lingg N, Bresolin I T L , et al. J Biotechnol, 2020, 324(S): 100020 10.1016/j.btecx.2020.100020 34154739

[48] Müller E, Sevilla M, Endres P. J Sep Sci, 2020, 43(12): 2255 32160397 10.1002/jssc.201900895 PMC7318155

[49] Fekete S, Veuthey J L, Beck A , et al. J Pharmaceut Biomed, 2016, 130(S1): 3 10.1016/j.jpba.2016.04.004 27084526

[50] King C, Patel R, Ponniah G , et al. J Chromatogr B, 2018, 1085: 96 10.1016/j.jchromb.2018.03.049 29649755

[51] Ma Y Y, Zhang D, Li Y R , et al. Food Science, 2023, 44(9): 185

[52] Wang S J, Xue Y X, Jin H B , et al. Modern Chemical Research, 2022(4): 1

[53] Li M T, Zhang Q L, Lin D Q , et al. J Chromatogr B, 2019, 1134/1135: 121850 10.1016/j.jchromb.2019.121850 31783251

[54] Lin D Q, Tong H F, Wang H Y , et al. J Phys Chem B, 2012, 116(4): 1393 22214397 10.1021/jp206817b

[55] Lin D Q, Tong H F, Wang H Y , et al. J Chromatogr A, 2012, 1260: 143 22975355 10.1016/j.chroma.2012.08.080

[56] Boschetti E. Trends Biotechnol, 2002, 20(8): 333 12127280 10.1016/s0167-7799(02)01980-7

[57] Wu M B, Zhang F F, Liang Y F , et al. J Dairy Sci, 2015, 98(5): 2973 25747828 10.3168/jds.2014-9142

[58] Tong H F, Lin D Q, Pan Y, et al. Biochem Eng J, 2011, 56(3): 205

[59] Luo Y D, Zhang Q L, Yao S J , et al. J Chromatogr A, 2018, 1533: 77 29241957 10.1016/j.chroma.2017.12.018

[60] Ma N, Yao D X, Yang H , et al. Ind Eng Chem Res, 2018, 57(39): 13295

[61] Shi Q H, Shen F F, Sun S. Biotechnol Prog, 2010, 26(1): 134 19785039 10.1002/btpr.295

[62] Zhao G F, Peng G Y, Li F Q , et al. J Chromatogr A, 2008, 1211(1/2): 90 18947830 10.1016/j.chroma.2008.09.108

[63] Phottraithip W, Lin D Q, Shi F , et al. Biotechnol Bioprocess Eng, 2013, 18: 1169

[64] Ren J, Xiang X, Ji F L , et al. J Chromatogr B, 2021, 1179: 122652 10.1016/j.jchromb.2021.122652 34280681

[65] Tong H F, Lin D Q, Chu W N , et al. J Chromatogr A, 2016, 1429: 258 26747689 10.1016/j.chroma.2015.12.047

[66] Zou X J, Zhang Q L, Lu H L, et al. Chem Eng J, 2019, 368: 678

[67] Arakawa T, Tomioka Y, Nakagawa M , et al. Antibodies, 2023, 12(1): 15 36810520 10.3390/antib12010015 PMC9944463

[68] Chu W N.[PhD Dissertation]. Hangzhou: Zhejiang University, 2019

[69] Kaleas K A, Tripodi M, Revelli S , et al. J Chromatogr B, 2014, 969: 256 10.1016/j.jchromb.2014.08.026 25203722

[70] Pezzini J, Cabanne C, Gantier R , et al. J Chromatogr B, 2015, 976/977: 68 10.1016/j.jchromb.2014.11.020 25482011

[71] Wang J, Zhou J X, Gowtham Y K , et al. Biotechnol Progress, 2017, 33(3): 658 10.1002/btpr.2454 28248007

[72] Cabanne C. J Chromatogr A, 2015, 1393: 57 25805720 10.1016/j.chroma.2015.03.018

[73] Wan Y, Zhang T, Wang Y M , et al. Protein Expres Purif, 2020, 175: 105712 10.1016/j.pep.2020.105712 32738441

[74] Song M K, Yang H, Cao W T , et al. Process Biochem, 2023, 132: 110

[75] Bernardo S C, Carapito R, Neves M C , et al. Molecules, 2022, 27(5): 1618 35268719 10.3390/molecules27051618 PMC8911583

[76] Liu H F, Chen J, Chen M L , et al. Anal Chim Acta, 2023, 1274: 341496 37455089 10.1016/j.aca.2023.341496

[77] Capela E V, Bairos J, Pedro A Q , et al. Sep Purif Technol, 2023, 305: 122464

[78] Chen J, Tetrault J, Zhang Y Y , et al. J Chromatogr A, 2010, 1217(2): 216 19819462 10.1016/j.chroma.2009.09.047

[79] Nagase K, Kanazawa H. Anal Chim Acta, 2020, 1138: 191 33161981 10.1016/j.aca.2020.07.075

[80] Shaibie N A, Ramli N A, Faizal N D F M , et al. Macromol Chem Phys, 2023, 224(20): 2300157

[81] Nagase K. Adv Colloid Interface Sci, 2021, 295: 102487 34314989 10.1016/j.cis.2021.102487

[82] Nagase K, Ishii S, Ikeda S, et al. Sci Rep, 2020, 10(1): 11896 32719404 10.1038/s41598-020-68707-7 PMC7385495

[83] Nomoto D, Nagase K, Nakamura Y , et al. Colloid Surface B, 2021, 205: 111890 10.1016/j.colsurfb.2021.111890 34089966

[84] Rahman M, Wang J Z, Xia H J, et al. Chem Eng J, 2020, 391: 123561

[85] Guo D M, Xia Y R, ur Rahman M, et al. Chinese Journal of Chromatography, 2023, 41(12): 1045 38093534 10.3724/SP.J.1123.2023.09028 PMC10719812

